# Tubercular Exudations into the Lungs, Etc., Checked by Cod-Liver Oil

**Published:** 1850-07

**Authors:** 


### Part 4.—Selections.

### ARTICLE I.

*Tubercular Exudation into the Lungs (Phthisis Pulmonalis)—Diseased Aortic Valves---Musical Heart-Sounds---Ulceration in the Lungs checked by Cod-Liver Oil.*

J. H. Bennett, Prof. of Institutes of Medicine, Edinburgh, relates, in a clinical lecture, published in the Monthly Journal of Medical Science, Feb., 1850, the following interesting case: Patrick Barclay, æt. 15, admitted June 25th, 1849. His previous history indicated that he had been of scrofulous habit from infancy. He had attended school regularly until a week ago, but could not take much exercise on account of a sore leg, which originated twelve months previously, in a fall. His diet has, for a long time, been very poor. On the 18th, he was attacked with cough, and this has continued till admission. He also complains of dyspnœa, on exertion. On admission he is excessively emaciated. He complains of cough, which is sometimes very prolonged, but has no pain nor difficulty of breathing. The chest expands well on inspiration. Cough eaaily excited, and occasionally severe. Sputa viscid, frothy, and tinged with blood. On percussion, there is great dullness on the right side, especially under the clavicle; the left side is also dull to a slight extent. On auscultation, distinct bronchophony, loud friction râle, and mucous râle, approaching cavernous, are heard in the upper right side, in front; and these become more faint towards the lower part of the lung. On the left side, friction râles are also heard in the upper part in front. Behind, on the right side, vocal resonance, not so distinct, but râles the same as in front. Pulse 114, strong and sharp. The heart's apex beats below sixth rib; impulse increased, but percussion does not indicate lateral expansion. On auscultation, a chirping musical murmur is heard over the apex of the heart, at the end of the first sound. This murmur becomes much more faint towards the base. To the left of the manubrium of the sternum, a bellows murmur takes the place of the second sound. This murmur is quite concealed by loud friction râles, when respiration is going on, but is immediately perceived when the patient holds his breath. Tongue slightly furred : appetite good ; some thirst. Bowels regular; urine natural. Sp gr 1020 ; not coagulable. The chest, face, and arms are covered with an eruption of purigo, which he has had several times. On the right thigh, towards the lower part, there are several cicatrices and three sinuses, which communicate with dead bone. Is much troubled with sweating, which at night is very profuse. To have good diet, with sweet milk, morning, and evening, and a dessert-spoonful of Cod-Liver Oil three times a-day. ℞. Mist. scillæ ℥iv ; tinct. opii ammon. ℥ss ; aquæ font. ℥ij. M. Half-an-ounce, three times a-day. 30th. Friction râle less. Gurgling râle, on right side. Upper part of chest to be rubbed with tartar emetic ointment. July 2d. Chirping murmur has become faint, and occasionally is inaudible. Has vomited his food several times. Napthæ ʒiss, to be added to mixture: to have beer for drink. 5th. Chirping murmur quite gone. 8th. Chirping murmur returned. Cough severe—causing vomiting. Eruption, brought out by ointment, painful. Omit the ointment, and mixture. ℞. Pulv. tragacath. co. ʒi ; napthæ medic. ʒi ; sol. mur. morph. iij ; syrup aurantii ℥ss ; mist. scillæ ʒv. M. A table-spoonful thrice a day. 31st. A seton was introduced beneath the right clavicle. Still vomits in the morning, but takes food and medicine better. August 6th. The expiratory murmurs under right clavicle are now quite dry. Vomiting is diminished. Omit the mixture. ℞. Ferri citrat ʒss. ; tinct. aurantii et syrupi, āā ℥ss ; infus. columbœ vi. M. A table-spoonful three times a-day. 12th. The seton discharges freely, causing great irritation, and is to be withdrawn Sept. 7th. Appearance of patient much improved. Sounds of cavity in chest continue dry. Takes now again a table-spoonful of the Oil three times a-day. Oct. 28th. Musical murmur has entirely disappeared. He is becoming quite fat, and is able to go about the ward all day. Complains only of slight cough at night, and palpitation on exertion. The right infra-cleviclar region is becoming flat. Omit the mixture, and also the Cod-Liver Oil. Nov. 18th. Cough has returned with slight mucous expectoration ; and, on auscultation, mucous and sibilant rales are heard over the chest. Ordered to recommence the Oil. ℞. Mist scilloe ℥vss ; vini ipecac. ʒij ; sol. mur. morph., ʒi. M. A table-spoonful three times a-day.

From this time he rapidly improved. The cavity became perfectfully dry, and respiration over it was accompanied by blowing murmurs. Cough and expectoration greatly diminished. His general appearance is healthy, and he is very stout. On Jan. 13th, it is noted that, on percussion, a distinct sound is heard in the right infra-clavicular region, and faintly also on the left side. On auscultation, the heart's sounds are loud all over the chest, the second sound being accompanied with a distinct bellows murmur. Musical murmur has never returned. There are broncophony and prolonged expiration in the right infra-clavicular region, but no moist sounds. Sleeps well, and is very little troubled with cough. Does not sweat; is very fat; appetite good. This boy, as far as all general symptoms are concerned, may be regarded as having been in good health for the last two months.

This case offers a good instance of the advantages of Cod-Liver Oil. On admission, he presented the wasting characteristics of Phthis in its last stage. The emaciation was extreme. The cough and sweating most distressing; and the physical signs demonstrated a cavity as large as the fist, in the right lung. Under the use of the Oil, his strength rallied. After a time, it was given up, on account of his becoming so fat. Gurgling ráles, and other signs of exudation, however, once more became apparent, and again disappeared when the use of the Oil was resumed. He has continued to take it ever since, and the cavity is evidently contracting. With the exception of very slight cough, and expectoration in the morning only, he may be considered as enjoying good general health. The sequel of this case will be given on a future occasion.—*Amer. Jour. Med. Science.*

